# Mining drug–target and drug–adverse drug reaction databases to identify target–adverse drug reaction relationships

**DOI:** 10.1093/database/baab068

**Published:** 2021-10-22

**Authors:** Cristiano Galletti, Patricia Mirela Bota, Baldo Oliva, Narcis Fernandez-Fuentes

**Affiliations:** Department of Biosciences, U Science Tech, Universitat de Vic-Universitat Central de Catalunya, Carrer Laura 13, Vic, Catalonia 08500, Spain; Department of Biosciences, U Science Tech, Universitat de Vic-Universitat Central de Catalunya, Carrer Laura 13, Vic, Catalonia 08500, Spain; Department of Experimental and Health Sciences, Structural Bioinformatics Group, Research Programme on Biomedical Informatics, Universitat Pompeu Fabra, Barcelona, Catalonia 08003, Spain; Department of Experimental and Health Sciences, Structural Bioinformatics Group, Research Programme on Biomedical Informatics, Universitat Pompeu Fabra, Barcelona, Catalonia 08003, Spain; Department of Biosciences, U Science Tech, Universitat de Vic-Universitat Central de Catalunya, Carrer Laura 13, Vic, Catalonia 08500, Spain

## Abstract

The level of attrition on drug discovery, particularly at advanced stages, is very high due to unexpected adverse drug reactions (ADRs) caused by drug candidates, and thus, being able to predict undesirable responses when modulating certain protein targets would contribute to the development of safer drugs and have important economic implications. On the one hand, there are a number of databases that compile information of drug–target interactions. On the other hand, there are a number of public resources that compile information on drugs and ADR. It is therefore possible to link target and ADRs using drug entities as connecting elements. Here, we present T-ARDIS (Target—Adverse Reaction Database Integrated Search) database, a resource that provides comprehensive information on proteins and associated ADRs. By combining the information from drug–protein and drug–ADR databases, we statistically identify significant associations between proteins and ADRs. Besides describing the relationship between proteins and ADRs, T-ARDIS provides detailed description about proteins along with the drug and adverse reaction information. Currently T-ARDIS contains over 3000 ADR and 248 targets for a total of more 17 000 pairwise interactions. Each entry can be retrieved through multiple search terms including target Uniprot ID, gene name, adverse effect and drug name. Ultimately, the T-ARDIS database has been created in response to the increasing interest in identifying early in the drug development pipeline potentially problematic protein targets whose modulation could result in ADRs.

Database URL: http://www.bioinsilico.org/T-ARDIS

## Introduction

One of the main major problems faced in drug development is the lack of toxicology or safety information for targets (1). This fact results in a high level of attrition of drugs entering clinical trials due to the severity of adverse drug reactions (ADRs) associated with toxicity, significantly increasing the costs and therefore limiting the development of novel drugs for emerging targets (2). One of the most conventional methods in past years relied on the use of animal models. However, animal models imply high maintenance cost and ethical drawbacks and not always transferable to human biology (3), and thus computational approaches can provide useful predictions.

There are a number of approaches that can be used to decrease the risk associated with the development of novel drugs from a drug-centric point of view. *In-silico* approaches have demonstrated their utility in estimating the toxicity of drug candidates, exploiting features such as composition, structure and binding affinity. These methods include various examples of machine learning and deep learning (4). Other studies are based on target-based predictions, analyses of the underlying protein network and interactions and quantitative structure–activity relationships. The latter have been used to model numerous drug safety endpoints including drug lethal dose of 50%, the so-called LD50 values, skin/eye irritation and tissue-specific toxicity, making it one of the most used parameters for estimating the toxicity of a drug (5). The use of curated protein target sets, conforming so-called safety panels, are also used to assess the potential liability of novel drugs during pre-clinical stages (6). Finally, information about potential liability of drugs can be also obtained post-development in the context of pharmacovigilance including a number of approaches that mine information for a range of databases such the Food and Drug Administration (FDA) spontaneous reporting systems database (5, 7, 8).

All the methods presented above are drug-centric, i.e. the prediction of potential ADR is based solely on the properties of the drug but not on the putative or known protein targets. In fact, while there are well-established methodologies and resources, as shown above, to associate drugs to ADR, it is less so to associate ADR to protein targets. Examples of the latter include the ADReCS-Target database (9) and a recent study on ADRs compiled from clinical trials and post marketing reports (10). A different take on the issue would be to identify the link between ADR and proteins, using drugs as a connecting element. In principle, the idea is very straightforward: if drug X causes ADR Y and drug X binds to protein Z, then protein Z is related to ADR Y. This simple statement is, however, incorrect. As pointed out by Kuhn and colleagues (11), most drugs bind to sets of pharmacologically similar proteins, for example, members of the same protein family. While it is likely that only one of the targets is responsible for a given ADR, a direct Target–ADR association, as in this simple approach, would relate each target to each possible ADR of the same drug, creating erroneous or non-existent relationships, i.e. false positives. This association needs to be validated statistically, and the method described by Kuhn *et al.* (11) provides a defined path identify statistically significant associations between ADR and proteins using drugs as the connecting elements.

T-ARDIS (Target—Adverse Reaction Database Integrated Search), the database presented here, contains statistically validated associations between protein targets and potential ADR derived from the association drug–ADR and drug–protein. In the first stage, drug–ADR and drug–protein associations were mined from different databases. In the case of drug–protein, the databases included the Drug–Target Commons (12) and STITCH (13) databases. Drug–ADR associations were mined from FDA Adverse Event Reporting System (FAERS) (14), MEDEFFECT (15), SIDER (16) and OFFSIDES (17). Upon mining, by parsing and filtering these databases, the associations between proteins and ADRs were established using the method described by Kuhn *et al.* (11) as described above. The results are therefore a number of protein–ADR associations that are statistically significant and that can be of use as complement to other approaches to identify potential liabilities associated with protein targets.

Currently, T-ARDIS compiles over 3000 ADRs associated with over 200 proteins. Users can easily access the data searching by the drug name (common name), type of ADR as defined in MedDRA dictionary (18) or the protein UNIPROT (19) identification code or gene name. The results are returned in a tabular from listing the principal descriptor for each entry such as the drug name, the target UniProt ID, gene name, the MedDRA classification for ADR, together with the results of the statistical validation (*P*-value of association and its correction for multiple testing, *q*-value, including the contingency table used). Moreover, it will be possible to access external links to the native drug target or drug–ADR database, together with related repositories.

## Material and methods

### Databases containing drug–ADR information

Four different databases were parsed and mined to identify drug–ADR associations: OFFSIDES (17), SIDER4.1 (16), MEDEFFECT (15) and FAERS (14). OFFSIDES is a manually curated database available at http://tatonettilab.org/resources/nsides/. SIDER4.1 is a database of drugs, ADR and indications mined from the FDA drug labels. The version used in this study is SIDER4.1 released 21 October 2015 available at http://sideeffects.embl.de/. The FAERS or AERS is a centralized pharmacovigilance database developed to integrate the U.S. FDA’s post marketing safety surveillance program. The data stored in this database represent one of the major repositories regarding drug–ADR relationships, although it requires a curation before that can be used (see below ‘Curation of FAERS database’). The version included in T-ARDIS was last updated in March 2020 and is available at: https://fis.fda.gov/extensions/FPD-QDE-FAERS/FPD-QDE-FAERS.html. Finally, the MEDEFFECT, Canada’s sister database of the FAERS. Adverse reaction reports are submitted by consumers and health professionals, who submit reports voluntarily, and manufacturers and distributors (also known as market authorization holders), who are required to submit reports according to the Canadian Food and Drugs Act. The version of MEDEFFECT included in T-ARDIS was updated in May 2020 and is accessible at https://www.canada.ca/en/health-canada/services/drugs-health-products/medeffect-canada/adverse-reaction-database/canada-vigilance-online-database-data-extract.html.

The adverse event report descriptions are coded as medical terms as defined in the MedDRA vocabulary and ontology (18). The entries in MedDRA are reported using five hierarchical levels of medical terminology, ranging from a very general System Organ Class (SOC—e.g. gastrointestinal disorders) term to a very specific Lowest Level Term (e.g. feeling queasy). Each term is linked to only one term on a higher level. For each drug–ADR database, we manually checked that all adverse reactions were registered as MedDRA Reaction terms at Preferred Term (PT) level that describes a single medical concept. We also used the SOC definition of MedDRA to filter unspecific ADR (see the ‘Filtering of ADR based on SOCs’ section).

### Curation of FAERS and MEDEFFECT databases

Prior to using the data present on the FAERS database, a curation of the records was performed. This step is required due to the heterogeneity in the reports as these are uploaded directly by health-care professionals (physicians, pharmacists, nurses and others) and other actors (patients, family members, lawyers and others.) Thus, the quality of the reports varies substantially and there are often typos (e.g. misspelled drug names), missing information and other errors. To obtain a curated and standardized version of FAERS and MEDEFFECT, we relied on a modified pipeline specially developed for the standardization of FAERS records (20) and adapted to MEDEFEECT. In particular, this pipeline uses standardized vocabularies with drug names mapped to RxNorm concepts (21) and exploits the demographic information on the patients in order to remove duplicates. To identify statistically significant associations between drugs and ADRs, the method proposed by Huang *etal*. (22). was applied to the resulting databases originating from the standardization pipeline described above. Finally, only those drug–ADR associations that are statistically significant, i.e. the likelihood ratio value is above the 5th percentile of the multinomial distribution, and present both in FAERS and MEDEFFECT were kept.

### Filtering of ADR based on SOCs

Some of the ADRs reported are very general or not specific to body parts, tissues or underlying human biology. For this reason and as described in (23), any ADR belonging to the following SOCs were discarded.

#### General disorders and administration site conditions

As the name suggests, this SOC contains terms that do not readily fit into the hierarchy of any one SOC or are non-specific disorders that impact several body systems or sites. To be noted that representing PTs in this SOC in each potential secondary SOC would create an inordinately large number of redundancies. Therefore, most of the PTs in this SOC are primarily linked to SOC General disorders and administration site conditions and have limited representation in secondary SOCs (e.g. PT Injection site atrophy is primarily to SOC General disorders and administration site conditions and secondarily only to SOC injury, poisoning and procedural complications).

#### Injury, poisoning and procedural complications

This SOC provides a grouping for those medical concepts where an injury, poisoning, procedural or device complication factor is significant in the medical event being reported. As a general rule, in this SOC all the events appear directly attributed to trauma, poisoning and procedural complications, in other words, all the events due to an external cause.

#### Investigations

For MedDRA, an ‘investigation’ is a clinical laboratory test concept (including biopsies), radiologic test concept, physical examination parameter and physiologic test concept (e.g. pulmonary function test). Only PTs representing investigation procedures and qualitative results (e.g. PT blood sodium decreased, PT blood glucose normal) appeared in this SOC. Terms representing conditions (e.g. hyperglycemia) or mixed concepts of conditions with an investigation are excluded from this SOC and can be found in the respective ‘disorder’ SOCs (e.g. PT hyperosmolar state, PT haemosiderosis, PT orthostatic proteinuria and PT renal glycosuria).

#### **Neoplasms benign, malignant and unspecified** (**incl.cysts and polyps**)

This SOC is classified anatomically, with pathologic sub-classifications for staging of both benign and malignant neoplasms.

#### Product issues

This SOC includes terms relevant for issues with product quality, devices, manufacturing quality systems, product supply and distribution and counterfeit products.

#### Social circumstances

The purpose of this SOC is to provide a grouping for those factors that may give insight into personal issues that could have an effect on the event being reported. Essentially, this SOC contains information about the person, not the adverse event. As an example, terms such as PT drug abuser and PT death of relative are found in this SOC.

#### Surgical and medical procedures

This SOC contains only those terms that are surgical or medical procedures. The nature of this SOC makes it more of a ‘support’ SOC for recording case information and for developing queries.

#### Infections and infestations

This SOC just provides information on location linked to infectious disorders but not to specific targets.

#### Psychiatric disorders

The following high-level general terms and high-level terms were excluded from this specific SOC due to being too general and/or broad. These included the terms: depressed mood disorders and disturbances; eating disorders and disturbances; impulse control disorders not elsewhere classified (NEC); manic and bipolar mood disorders and disturbances; personality disorders and disturbances in behaviour; psychiatric disorders NEC; suicidal and self-injurious behaviours NEC; paraphilias and paraphilic disorders and sexual and gender identity disorders NEC.

### Databases containing drug–protein information

Two different databases were used to extract drug–protein associations. These include Drug-Target Commons (DTC) database (https://drugtargetcommons.fimm.fi) (12). The DTC aims at providing an open-data platform for a community-driven crown-sourcing effort to annotate drug–target associations and provides information on drugs’ bioactivity such IC50, EC50 and potency values. The version included in T-ARDIS was downloaded in April 2021 from https://drugtargetcommons.fimm.fi. The second database considered was STITCH (13). STITCH provides a complementary view on drug–target associations as it relies on different sources of information combined into a composite scoring function (24). The version included in T-ARDIS is 5.0 and is accessible at http://stitch.embl.de.

The starting databases were subjected to two filter steps to ensure that biologically/therapeutically relevant associations are captured and that redundant entries originating from the same drug been named differently. The Uniprot ID was used to ensure that the target was the same in both databases. DTC provide already this information for each pair drug–target but in the case of STITCH the Uniprot ID was retrieved programmatically from the Uniprot database (19) using the STRING (25) identification code. In the case of DTC, only drug–protein association with a reported IC50 (or EC50) of 100 nM or better was considered. In the case of the STITCH database, a cut-off of 0.8 was applied, thus only association with a better score was considered. To avoid redundancy, the drug entries were unified using the InChiKey hash descriptors and the drug’s standard name ensuring that not redundant entries appear in the consolidated dataset.

### Statistical association protein–ADR using drug–protein and drug–ADR relationships

**Figure 1.**
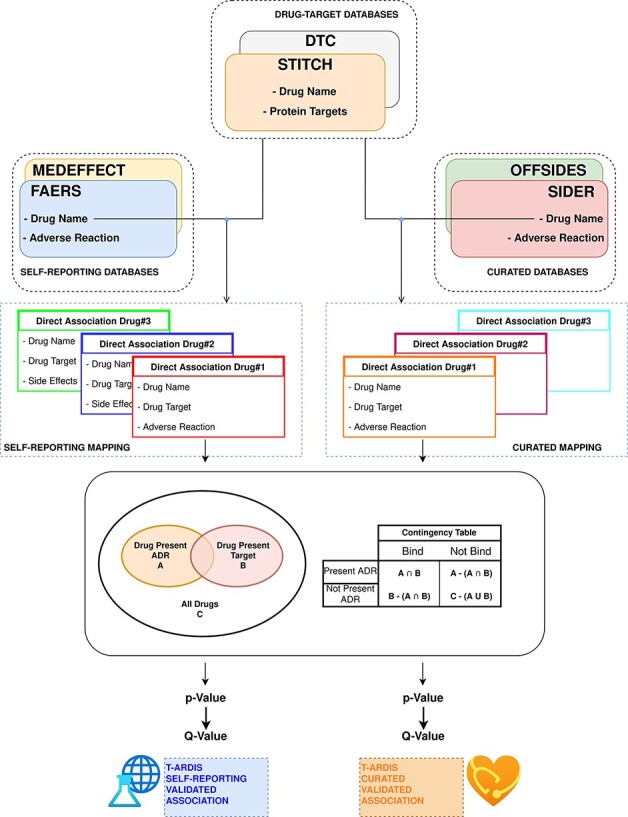
Workflow followed to combine and derive statistical associations between proteins and ADR. Drug–ADR and drug–target associations are retrieved from relevant databases. Subsequently, statistical association between proteins and ADRs is computed as described by Kuhn *et al.* (10).

The statistical significance of ADR–protein associations was calculated following the method proposed by Kuhn *et al.* (11). In a nutshell, the method computes a contingency matrix for each ADR–protein pair and calculates the *P*-value using Fisher’s exact test. The elements of the contingency matrix are as follows: (i) the number of drugs that present the given ADR; (ii) the number of drugs that binds to the given protein; (iii) the number of drugs that both present the given ADR and bind to the given protein and (iv) how many drugs neither present the ADR nor bind to the given target. Given the high number of relationships, *P*-values were corrected for multiple testing using the ‘*q*-value’ module contained in the python package ‘MultyPy’ (26). An ADR–protein relationship was accepted if the computed *q*-value is equal or smaller than 0.05. Figure 1 shows an outline of this annotation approach, from the mining of individual databases to statistical association.

Prior to the calculation of protein–ADR statistical associations, the drug–ADR databases were divided in two different sets: curated and self-reporting drug–ADR association. The curated included drug–ADR associations extracted from SIDER and OFFSIDES, while the self-reporting set included drug–ADR association from FAERS and MEDEFFECT. The logic follows on distinguish between these two groups as the origin of information is very different as mentioned above. Therefore, the statistical associations between protein–ADR present in T-ARDIS originate from any of these two sets as the drug–target associations are common to both, i.e. DTC and STITCH databases. The unifying entity between drug–protein and drug–ADR is of course the drug entity, and the unification between both groups was done the using the drug’s standard name. To make sure an unequivocal association, a Tanimoto 2D chemical similarity score was computed with a cut-off of 0.7 using the Rdkit Conda package (27). Finally, drugs presenting less than 10 ADRs were also discarded.

In the case of the drug-target databases, a filtering procedure was implemented as described in Kuhn *et al.* (11). First, proteins related to drug metabolism were discarded. These were selected using the Gene Ontology annotation (28), and thus proteins belonging to GO terms: GO:0042737 (drug catabolic process) and GO:0017144 (drug metabolic processes) were discarded. Second, a sequence similarity filter was implemented to remove highly redundant proteins using CD-HIT (29) at 90% sequence identity cut-off. A subsequent clustering step was devised to group proteins into families using a sequence identity cut-off of 70% and families with more than 10 members for same drug were excluded preserving just the association with the centroid of the cluster. Finally, as discussed in Kuhn *et al.* (11), for each of the protein–ADR groups, the main target was identified as reported (30) and the rest of the members of the group were kept if sharing at least 50% of the drugs binding to the main target.

### Benchmarking datasets

Four different datasets were used to compare the associations uncovered by T-ARDIS. The first set was extracted from the ADReCS-Target database (9) from which 1710 protein–ADR top scoring associations were compiled. The second set derives from the recent wok by Smit *et al.* (10) that albeit containing an older release of SIDER (ver.3) was used to extract circa 2000 protein–ADR associations. The third set relates to a set of 225 pairwise interactions validated in the work of Kuhn *etal.* (11). Finally, the fourth set is a manually curated set mined for scientific publications presented in the work by Kuhn *et al.* (11), which includes 816 protein–ADR associations (Table 1).

**Table 1.** Comparison of different datasets and T-ARDIS

SET	# Associations	Self-reporting^a^	Curated^b^
Associations mined from the literature in Kuhn *et al.* (11)	224	27 (4)	17 (6)
Associations validated in vivo in Kuhn *et al.* (11)	2170	115 (69)	113 (85)
Associations described in Smit *et al.* (10)	2153	340 (48)	297 (167)
Associations from ADReCD-Target database (9)	816	171 (14)	87 (11)

a Associations present in the self-reporting set of T-ARDIS; significant associations shown within parentheses (*q*-values < 0.05).

b Associations present in the curated set of T-ARDIS; significant associations shown within parentheses (*q*-values < 0.05).

## Results

### Combining different databases increases the coverage of associations

**Figure 2.**
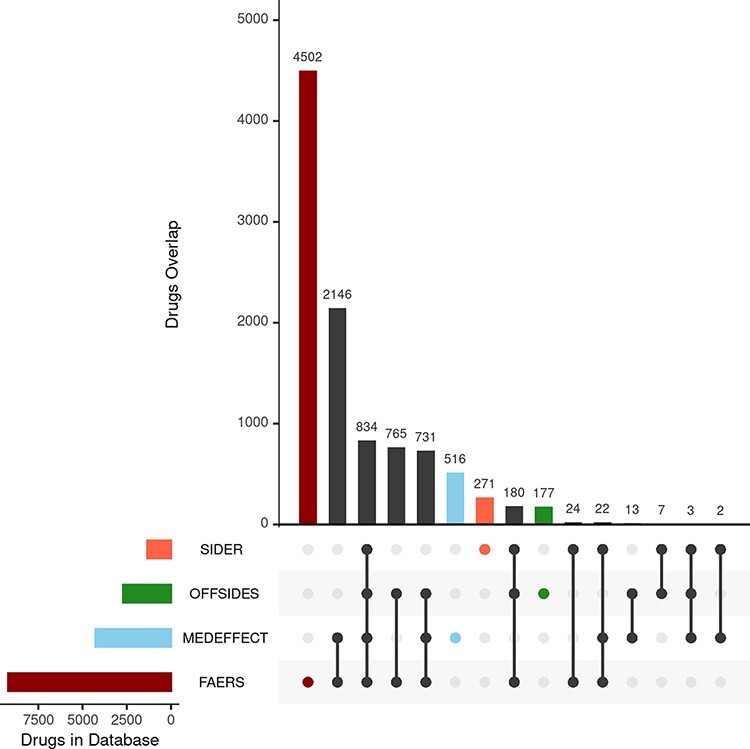
Upset plot showing the overlap between the different databases compiling drug–ADR associations. FAERS, MEDEFFECT, OFFSIDES and SIDER represented as dark red, light blue, green and orange, respectively.

We first consider the databases with drug–ADR associations. As described in the ‘Materials and methods’ section, the nature and purpose as well as the level of curation of these databases vary. There is a core of drug–ADR associations, which are common to all databases (Figure 2). The overlap between OFFSIDES and FAERS databases is relatively high and expected as drug–ADR associations annotated in OFFSIDES are subsequently added to FAERS on new releases. FAERS and MEDEFFECT rely on multiple sources and spontaneous reporting systems and contain the largest number of drugs–ADRs associations as well as the largest percentage of unique entries. Following the curation approach, over 4 million pairwise interactions originating from over 9000 compounds and around 17 000 unique ADR were obtained from FAERS. In the case of MEDEFFECT, 1.5 M drug–ADR associations were uncovered from a total of over 4000 and 12 000 drugs and ADR events annotated in the database, respectively.

Unlike FAERS and MEDEFFECT, SIDER and OFFSIDES contain manually curated associations of drugs and ADRs. These databases have a lower number of associations when compared to spontaneous reporting databases FAERS and MEDEFFECT (between 1 and 2 orders of magnitude less). In the case of SIDER, over 108 000 pairwise interactions were mined for a total of 1344 unique drugs and 2303 ADRs. OFFSIDES yielded a large number of pairwise drugs–ADR associations: 1.5 M associations from a total of 2708 and 4368 unique drugs and ADRs. In terms of uniqueness of information, FAERS and MEDEFFECT show a larger percentage of shared drugs between the different databases (Figure 2).

The second group of databases considered were those describing drug–protein target associations including DTC (12) and STITCH (13). The nature of both databases is rather different and so it is reflected in the number of associations extracted from each individual database. In the case of STICH, over 10 000 drug–target associations were retrieved after applying the filter described in the ‘Materials and methods’ section accounting for 5007 and 1075 different drug and chemical compounds and proteins (as per Uniprot IDs). respectively. In the case of STITCH, the number of associations was much larger: over 6 M from over 42 000 chemical compounds (including approved drugs) and 7264 different proteins. The overlap between both databases in terms of shared drugs was around 1600.

### Proteins–ADR relationship from mined drug–ADR and drug–protein associations

After curation of drug–target and drug–ADR database and filtering, the associations between proteins and ADRs were obtained. The association was based on the drug entities shared among the databases. It is important to stress that self-reporting (FAERS and MEDEFFECT) and curated (OFFSIDES and SIDERS) drug–ADR sources of information were not combined but treated independently. In the case of protein–ADR associations uncovered from combining drug–target and drug–ADR (self-reporting), a total of 998 drugs were mapped unequivocally on both sets (i.e. drug–target, drug–ADR) yielding over 100k statistically significant (i.e. *q*-value ≤ 0.05) protein–ADR associations accounting for around 3k and 211 different ADRs and proteins, respectively. In the case of the second group of drug–ADR databases, the curated set (or not self-reporting), i.e. SIDER and OFFSIDES, a total of 1135 common drug entities were identified between drug–target, yielding circa 40k statistically significant associations protein–ADR including 537 and 194 ADRs and proteins, respectively.

**Figure 3.**
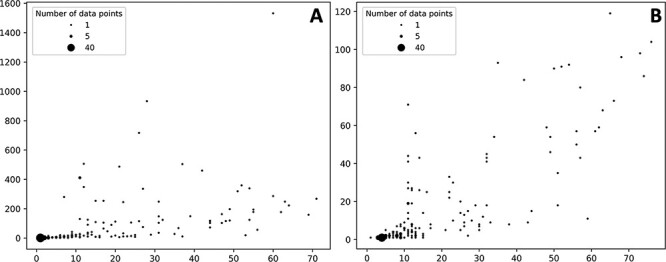
Bubble plots showing the number of drugs per protein (*X* axis) vs number of statistically significant ADR per protein (*Y* axis). (A) Distribution of the self-reporting set; (B) distribution of the curate set. Refer to the ‘Material and methods’ section for the description of self-reporting and curated sets.

The number of ADR associated with a given protein target varies but in most cases the number of associated ADR to proteins is low both in the case of data extracted from the self-reporting and curated dataset (Figure 3). As expected, the number of associated ADRs to a given target relates to the number of drugs identified to target the given protein; as the number increases, the number of ADRs also increases, albeit with a clearer trend in the case of the curated dataset (Figure 3B). Nonetheless there are a number of proteins associated with a large number of ADRs. In the case of the protein–ADR associations uncovered from the self-reporting dataset proteins, interleukin-8 (Uniprot ID: P10145), endothelin-1 (Uniprot ID: P05305) and leptin (Uniprot ID: P41159) were associated with 1532, 933 and 717 ADRs, respectively. In the case of the curated dataset, the figures are smaller and among the top three proteins are the 5-hydroxytryptamine receptor 2C (Uniprot ID P28335), the 5-hydroxytryptamine receptor 1A (Uniprot ID: P08908) and the alpha-2A adrenergic receptor (Uniprot ID: P08913) with 119, 104 and 98 associated ADRs, respectively. The explanation to this high number relates to the biological role played by these proteins. For instance, leptin is associated with over 150 biological processes (as per GO classification) ranging from signal transduction (GO:0007165) to autophagy regulation (GO:0010507). Moreover, the distribution of the number of ADR per target is in line with the work presented by Kuhn *et al.* (11) where the statistical association approach was described and that is the basis of T-ARDIS.

### T-ARDIS associations complement those of other resources

Association between ADRs and proteins uncovered in T-ARDIS were compared to previous works to assess the level of agreement and complementarity. The overall representation of target–ADR associations described in these four datasets, i.e. regardless of whether significant or non-significant, is low (Table 1). For instance, in the case of the set A (target–ADR associations mined from the literature), only 12% and 8% are presented in the self-reporting and curated sets of T-ARDIS, respectively. Overall, the values range from 20% to 5% in the case of self-reporting set and from 8% to 5% in the case of the curated set. These relatively low values can be due to two different causes. On the one hand, the lack of target–ADR associations in T-ARDIS can be due the fact that no safety issues have been reported either in self-reporting (FAERS, MEDEFFECT) or curated databases (OFFSIDES, SIDER). It could also be that association between the given drug and target is not present in any of the following two databases used in this study: DTC and STITCH. On the other hand, and as described in the ‘Methods’ section, a robust and stringent procedure is followed when compiling and integrating the databases used to derive T-ARDIS. Thus, the given drug–ADR and/or drug–target association can be present but do not succeed to pass the filtering steps. In any case, these results come to illustrate the complementary nature of T-ARDIS to that of other resources available in the field and thus achieving a more comprehensive and complete view of target–ADR associations.

### Examples of uncovered associations

Examples of protein–ADR associations uncovered by the approach presented here have been confirmed in the literature. For example, the cyclo-oxygenase 2 enzyme found in the gastric mucosa (COX-2 or PTGS2; Uniprot ID: P35354) is inhibited by the anti-inflamatory drug aspirin (acetylsalicylic acid). The aspirin also acts against the prostagladin G/H synthase 1 (COX-1 or PTSG1; Uniprot ID: P23219) (31, 32). These secondary interactions may be the concomitant cause for gastritis and bleeding ulcer as mentioned in various publications even since 1955 (33, 34). In our analyses, both PTGS1 and PTGS2 proteins are linked to Peptic ulcer and Peptic ulcer haemorrhage ADRs with significant *q*-values.

The sodium-dependent serotonin transporter (SLC6A4; Uniprot ID P31645) is inhibited by the serotonin norepinephrine reuptake inhibitor Venlafaxine, which in turn has been associated with sexual-dysfunction (35). In our analyses, SLC6A4 appears highly significantly associated (i.e. *q*-value << 0.05) with a range of different sexual dysfunctions (e.g. ejaculation failure and female sexual dysfunction).

Another example is illustrated by Budesonide and the glucocorticoid receptor (Uniprot ID: P04150). Identified ADRs to budesonide treatment include respiratory infections, coughs and headaches in the case of the inhaled form and tiredness, vomiting and joint pains in the oral form. A much rarer condition, adrenal insufficiency, has been identified in the case of the long-term use of the oral form of budesonide (36), which in T-ARDIS appears as a potential ARDs associated with the glucocorticoid receptor with a highly significant *q*-value. Furthermore, the association between glucocorticoids and adrenal insufficiency is an active topic of discussion in the current literature (37).

The activation of the 5-hydroxytryptamine receptor family (HTR1A, HTR1B and HTR1E; Uniprot IDs: P08909, P28222, and P28566, respectively) by zolmitriptan is reported to cause hyperaesthesia. In our analysis, the association between these proteins and hyperaesthesis were all significant, with *q*-values of 0.0001, 0.006 and 0.02 for HTR1A, HTR1B and HTR1E, respectively. It is worth mentioning that this association was identified and validated in vitro by Kuhn *et al.* (11). Overall, these examples, by no means a representative sample, show the usefulness of the data presented here that can be of use to identify potential liabilities associated with the targeting of proteins.

### Accessing and querying T-ARDIS

**Figure 4.**
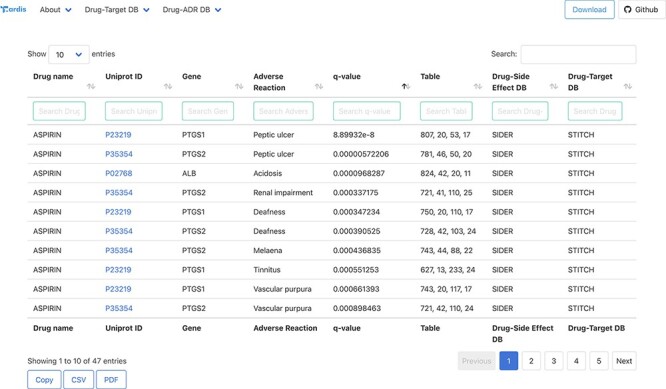
Snapshot of the result page example upon querying by drug ‘Aspirin’.

All the association between drugs–proteins including the original sources, i.e. drug–protein and drug–ADR, has been deposited and compiled in a biological database: T-ARDIS. T-ARDIS is available at: http://bioinsilico.org/T-ARDIS. T-ARDIS provides a convenient and easy access to the information including the option of searching and filtering associations based on tailored queries. The database is searchable by protein (Uniprot ID or gene name), drug or ADR name. The resulting tables provide information on the association between protein–ADR as well as the *q*-value of the association and parent databases, both drug–protein and drug–ADR (Figure 4). External links to native drug–target or drug–ADR databases, together with protein-related repositories, are also provided. Users also have the option to further filter the resulting table by querying by specific drug, ADR or parent databases (e.g. filtering those associations resulting from FAERS). The table can be also sorted by *q*-values, so most significant associations could be shown first. The tables can be downloaded in the different formats (simple copy, CSV or PDF). Finally, bulk downloads of the database and associated scripts to recreate the database are also available from the home page links.

## Discussion

Predicting associations between protein targets and ADR is desirable particularly in pre-clinical drug development in order to identify early in the process potential liabilities and toxicity-related aspects linked to proteins. Here, we present a fully automatic, large-scale, analysis to identify potential links between proteins and ADRs. By integrating public databases on drug–protein and drug–ADR associations, we have statistically identified significant relationships between protein and ADR using drugs as connecting elements. Highly significant associations, i.e. low *q*-values, are supported in the current literature and thus proving that uncovered associations could be useful as guiding evidence. The data compiled in this work have been deposited in a freely accessible database, T-ARDIS, which allows a convenient and easy access to the information. The mining of the databases, statistical inference and database updating is fully automatic and thus ensuring that data will be integrated as become available further facilitating our understanding of the mechanisms behind ADRs. We envisage that T-ARDIS represents a resource that will be useful to both academic and industry researchers working on drug development.

## Data Availability

All the data and scripts required to recreate T-ARDIS Database are available on GitHub at https://github.com/cristian931/Target—Adverse-Reaction-Database-Integrated-Search. The database is also available at http://bioinsilico.org/T-ARDIS.
